# Patient experiences with SARS-CoV-2: Associations between patient experience of disease and coping profiles

**DOI:** 10.1371/journal.pone.0294201

**Published:** 2023-11-20

**Authors:** Kathryn W. Hendrickson, Ramona O. Hopkins, Danielle L. Groat, Stephanie C. Stokes, Fiona M. Schroeder, Jorie M. Butler, Eliotte L. Hirshberg

**Affiliations:** 1 The Oregon Clinic, Department of Pulmonary, Critical Care, and Sleep Medicine East, Portland, Oregon, United States of America; 2 Department of Psychology and Neuroscience Center, Brigham Young University, Provo, Utah, United States of America; 3 Intermountain Health, Center for Humanizing Critical Care, Murray, Utah, United States of America; 4 Intermountain Health, Division of Pulmonary and Critical Care, Murray, Utah, United States of America; 5 Intermountain Health, Strategic Research, Salt Lake City, Utah, United States of America; 6 Department of Biomedical Informatics, University of Utah, Salt Lake City, Utah, United States of America; 7 Division of Geriatrics, Department of Internal Medicine, University of Utah, Salt Lake City, Utah, United States of America; 8 Informatics Decision-Enhancement and Analytic Sciences (IDEAS), Center for Innovation & Geriatrics Research, Education, and Clinical Center (GRECC), VA Salt Lake City Health Care System, Salt Lake City, UT, United States of America; 9 Division of Pulmonology, School of Medicine, University of Utah, Salt Lake City, Utah, United States of America; University of Macerata: Universita degli Studi di Macerata, ITALY

## Abstract

**Introduction:**

Severe acute respiratory syndrome coronavirus 2, (SARS-CoV-2,) caused an influx of patients with acute disease characterized by a variety of symptoms termed COVID-19 disease, with some patients going on to develop post-acute COVID-19 syndrome. Individual factors like sex or coping styles are associated with a person’s disease experience and quality of life. Individual differences in coping styles used to manage COVID-19 related stress correlate with physical and mental health outcomes. Our study sought to understand the relationship between COVID-19 symptoms, severity of acute disease, and coping profiles.

**Methods:**

An online survey to assess symptoms, functional status, and recovery in a large group of patients was nationally distributed online. The survey asked about symptoms, course of illness, and included the Brief-COPE and the adapted Social Relationship Inventory. We used descriptive and cluster analyses to characterize patterns of survey responses.

**Results:**

976 patients were included in the analysis. The most common symptoms reported by the patients were fatigue (72%), cough (71%), body aches/joint pain (66%), headache (62%), and fever/chills (62%). 284 participants reported PACS. We described three different coping profiles: outward, inward, and dynamic copers.

**Discussion:**

Fatigue, cough, and body aches/joint pains were the most frequently reported symptoms. PACS patients were sicker, more likely to have been hospitalized. Of the three coping profiles, outward copers were more likely to be admitted to the hospital and had the healthiest coping strategies. Dynamic copers activated several coping strategies both positive and negative; they were also younger and more likely to report PACS.

**Conclusion:**

Cough, fatigue, and body aches/joint pain are common and most important to patients with acute COVID-19, while shortness of breath defined the experience for patients with PACS. Of the three coping profiles, dynamic copers were more likely to report PACS. Additional investigations into coping profiles in general, and the experience of COVID-19 and PACS is needed.

## Introduction

Severe acute respiratory syndrome coronavirus 2 (SARS-CoV-2), the agent responsible for coronavirus disease 2019 (COVID-19), caused an influx of hospitalized patients with severe, acute disease world-wide [1]. Acute disease presentation can be asymptomatic or severe with multi-organ failure requiring treatment in the intensive care unit. Some patients, regardless of initial disease severity, go on to develop post COVID-19 syndrome (PACS, also referred to as post-COVID condition and long COVID) [2, 3] which effects them months to years after infection and requires ongoing care in the outpatient setting [4]. PACS is characterized by a variety of persistent symptoms [5] such as fatigue and cognitive impairment (including memory loss and concentration difficulties). In addition, body systems such as cardiac and pulmonary systems can be affected [6–8]. The lived experience of patients with persistent symptoms and how experience and symptoms might relate to one another is less well characterized.

PACS occurs in adults and children with probable or confirmed SARS-CoV-2 infection, which persists months to years and cannot be explained by an alternative diagnosis [9, 10]. The long term symptoms and duration of PACS are difficult to predict and not clearly associated with severity of acute illness [4]. Efforts to understand and treat PACS reveal that the syndrome affects each individual differently and risk factors are heterogeneous but may include older age, sex, and race and ethnicity [6]. PACS is classified as a chronic disease due to the duration of symptoms and wide-spread impact of PACS syndrome across race, gender, and age. In July, 2021, PACS with physical or mental symptoms lasting longer than 6 months which affect one or more aspects of daily activities is considered a disability under the Americans with Disabilities Act [11].

Individual experiences of disease are influenced by factors such as sex or behavior traits (coping styles) and such characteristics can be associated with a person’s experience of disease [12]. For example, men are more likely to test positive and be hospitalized with COVID-19, whereas women are more likely to self-report symptoms of PACS [13, 14]. Patient factors, like advanced age and comorbidities, are associated with an increased risk for severe, acute disease [15, 16]. Data suggest that some racial and ethnic groups may be differently impacted by PACS with, for example, a higher prevalence found in people of Hispanic/Latino ethnicity [17–19]. Understanding how individual factors impact the COVID-19 experience could be important to its treatment. The COVID-19 pandemic also revitalized interest in coping strategies [20–22]. Individual differences in coping styles and strategies used to manage COVID-19 pandemic related stress correlate with physical and mental health status (such as anxiety, depression and quality of life) [21, 23].

This diversity of presentation makes it crucial to incorporate patient reports to understand the experience and lasting effects of the disease. In addition, evidence based frameworks of stress and coping demonstrate that individuals under stress assess their stressful circumstances, harness internal resources, and engage in coping strategies to deal with stress [24]. The majority of research has focused on coping with the pandemic at large and not on coping with the experience of COVID-19 or PACS. Our study sought to address this gap and understand the relationship between COVID-19 symptoms, severity of acute disease (indicated by area of hospitalization) and coping profiles. In addition, we were interested if there are coping profile patterns that related to the lived experience of acute COVID-19 and/or PACS.

## Methods

In collaboration with our Patient Family Advisory Council (PFAC) we designed this study. Semi-structured interviews with concept elicitation were used to prompt symptoms experienced before, during, and after hospitalization as well as specific details of the COVID-19 experience, including what occurred during an average day in the hospital for hospitalized patients. Two cohorts of semi-structured interviews were completed. All semi-structured interviews were conducted 2–8 weeks after hospital discharge through a secure web platform including the participant and a member of the research team. A total of fifty-six patients who had a positive SARS-CoV-2 test and a diagnosis of COVID-19 viral illness completed the interviews. Interview exclusion criteria included prior cognitive impairment or significant mental illness impairing ability to participate in an interview, residence in a medical institution at the time of hospital admission, lack of stable domicile and/or not willing to share contact information, incarceration, and known or suspected pregnancy.

**Fig 1:**
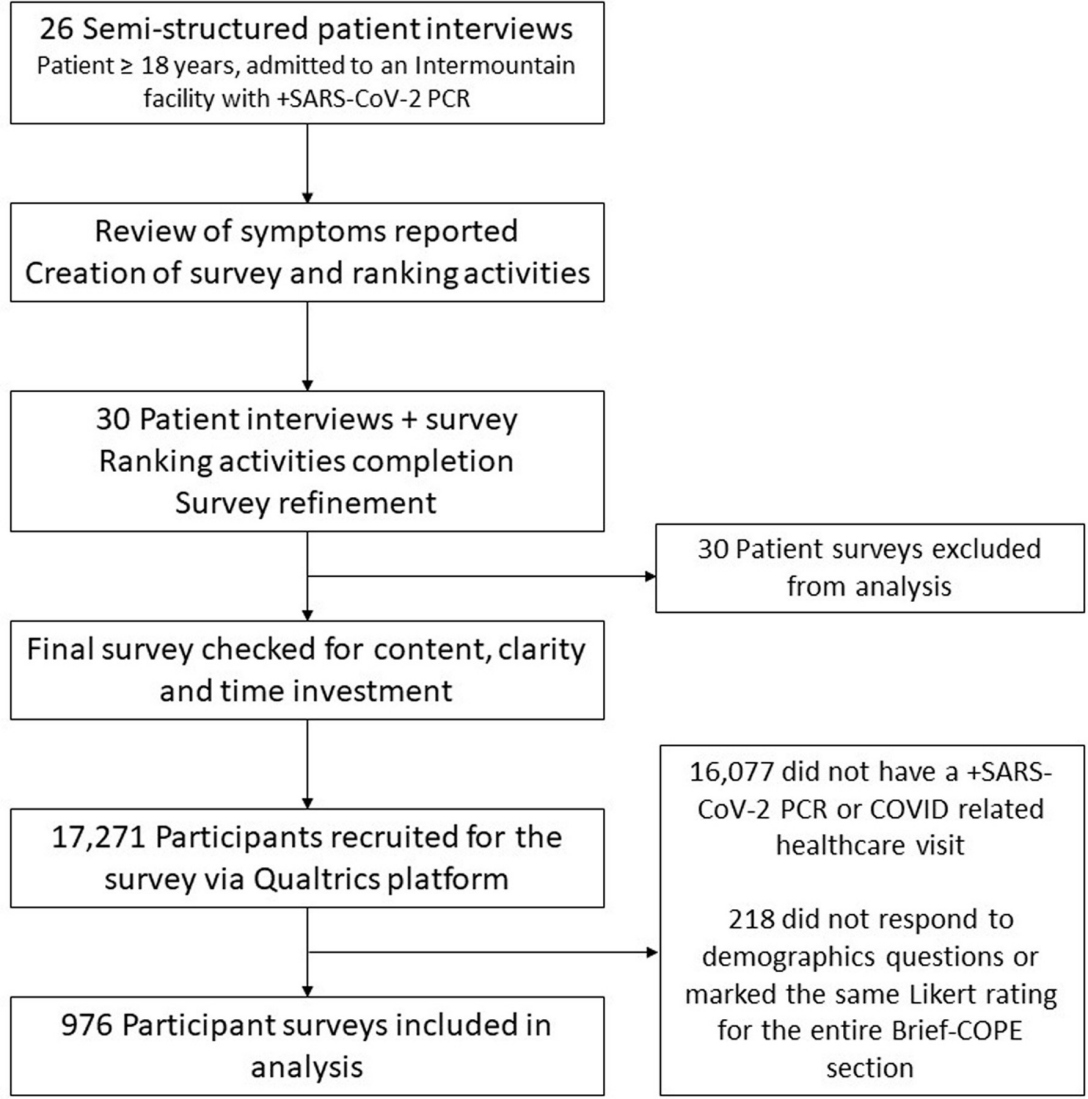
Study flow diagram.

Using the semi-structured interview data from the first 26 patients, we designed a survey instrument to assess symptoms, functional status, and recovery in a larger group of patients. This interview data was used to develop a ranking activity to allow patients to identify symptoms that they experienced, their most bothersome symptoms, and symptoms most important to be free from, and ranked a subset of symptoms by the degree to which the symptoms characterized their experience of COVID-19. The online survey also included validated questionnaires; the Brief-COPE to assess coping responses [25] and the Social Relationship Inventory (SRI) which was adapted in our prior work for clinical contexts in an effort to briefly assess complexity in social relationships [26, 27]. A second group of 30 patients **(Fig 1)** were recruited to complete the semi-structured interview and the online survey to evaluate the survey for completeness and clarity. Data from the second group was used to check the ranking questions that assess most important, common, and bothersome symptoms to make sure that the list generated from the data of the first group was not missing important symptoms and would reflect patient centered outcomes. We also assessed the time required to complete the survey to ensure the online survey was accessible and easy to navigate.

### Online survey and study population

Study patients were recruited from national research panels. An ethnically diverse group of COVID-19 survivors, aged 18–85 from around the United States, were recruited. The participants were screened and considered eligible if they reported a positive SARS-CoV-2 PCR and a COVID-19 related healthcare utilization (outpatient primary or urgent care, no hospital, inpatient hospital stay, or emergency department visit). Demographic data included age (grouped 10-year intervals (18–65+), and education level (less than high school, high school/GED, some college, associate degree, bachelor’s degree, master’s degree, doctorate/professional degree). The survey was administered using the Qualtrics platform, a nationally recognized consumer experience software. The online survey can be found in the Online Data Supplement **(ODS)**.

### Ethical considerations

The Intermountain Health Institutional Review Board approved the study and all procedures (IRB number 1051610). Informed consent was obtained for all participants prior to participating in the online, semi structured interviews. For the online survey, a cover letter of explanation was included at the start of the Qualtrics survey to inform potential participants that completing the survey implied consent. All data was securely stored using the Qualtrics secure web platform and then transferred to a secure REDCap system database [28, 29]. The interview participants received a $100 gift card as compensation for their time and effort. Survey participants received incentives through their contact with Qualtrics. The study was supported by Gilead contract number 2021029.

### Data and statistical analysis

Participants whose survey responses did not include answers to demographics questions, the Brief-COPE, or SRI questions were excluded from analysis. Participants that repeatedly marked the same answer for each question in the Brief-COPE section of the survey were excluded from analysis. Descriptive statistics were calculated as median (interquartile range) or count (%) as appropriate.

Cluster analysis, employing k-means clustering, was used to identify coping profiles based on patterns of responses to the strategies patients reported using to cope with COVID-19 related stress measured by the Brief-COPE, and quality of social support with person for whom they had close relationships was measured by SRI [30]. This analytic approach allows for identifying subgroups defined by similarities among multiple dimensions of interest, which is an improvement over examining coping strategies discretely [31]. To prepare the data for clustering analysis, the Brief-COPE and SRI responses were normalized between -1 and 1, thus weighting each question equally by the Euclidian distance calculation method used by k-means clustering. Silhouette analysis, which identifies the number of clusters with the lowest misclassification rate, was used to determine the optimal number of clusters, while maintaining adequate group size of no less than 15% of the participants. Once the optimal number of clusters was determined, the first two components of a principal component analysis were inspected to verify adequate separation between the groups, indicating unique coping profiles were indeed identified with the k-means clustering approach. We reviewed the emotional, behavioral, and social relationships of the clusters to describe them with a label. Descriptive statistics of self-reported demographics and symptoms were presented by coping profile and PACS status.

Chi squared test and Fisher’s exact test in cases where cell counts were less than 5, were used to test for differences between groups. Prior to performing statistical tests by PACS status and coping profiles, categories for age, gender, race/ethnicity, education, and religion were condensed into fewer categories. Statistical significance was set to 0.05. Due to the exploratory nature of the analyses, we did not correct for multiple comparisons. All statistical analyses were conducted using R 4.0.3.

## Results

### Demographics

We recruited a total of 17,271 individuals from the Qualtrics research panels. Of those, 1194 met study inclusion criteria. There were 218 (18%) of the respondents that did not interact with the survey in a meaningful way, resulting in 976 (82%) participants that were included in the analysis **(Fig 1).**

The majority of participants were ages 25 to 54 years (57%), and the most common education levels were high school/GED or some college (47%). Sixty one percent were female, 67% were non-Latino white and 16% were Black/African American with 12% Hispanic/Latino. Most (87%) respondents had health insurance. Fifty percent of participants did not require an interaction with a hospital facility, 27% visited the emergency department (ED), and 23% were admitted to the hospital (general hospital or intensive care unit [ICU]) **(Table 1).**

**Table 1:** Self-reported demographics, n = 976.

Attribute	Count (%)
Age, years	
18 to 24	124 (12.7%)
25 to 34	190 (19.5%)
35 to 44	184 (18.9%)
45 to 54	190 (19.5%)
55 to 64	156 (16.0%)
65 plus	132 (13.5%)
Sex	
Female	597 (61.2%)
Male	373 (38.2%)
Non-binary	6 (0.6%)
Race/Ethnicity	
White	657 (67.3%)
Black/African American	152 (15.6%)
Hispanic/Latino	116 (11.9%)
Asian	42 (4.3%)
Multiple/Other	8 (0.8%)
Not Reported	1 (0.1%)
Education	
Less than high school	28 (2.9%)
High school/GED	256 (26.2%)
Some college	213 (21.8%)
Associate Degree	102 (10.5%)
Bachelor’s Degree	226 (23.2%)
Master’s Degree	120 (12.3%)
Doctoral/Professional Degree	31 (3.2%)
Insurance	
Insured	850 (87.1%)
Care Level	
No Hospital	489 (50.1%)
Emergency Department	259 (26.5%)
Hospital Inpatient (includes ICU)	228 (23.4%)

The most common symptoms reported were fatigue (72%), cough (71%), body aches/joint pain (66%), headache (62%), and fever/chills (62%). The most bothersome or difficult (or most important) symptoms with the highest frequency were cough (38%), fatigue (32%), and body aches (30%). Twenty nine percent of our cohort reported experiencing PACS (which was described as long COVID on the survey) **(Table 2).**

**Table 2:** Self-reported symptoms.

Attribute	Count (%)
Symptoms over entire course of COVID-19 (multiple selections)	
Body aches/joint pain	642 (65.8%)
Brain fog	303 (31.0%)
Cough	689 (70.6%)
Chest pain	362 (37.1%)
Fatigue	702 (71.9%)
Fever/chills	608 (62.3%)
GI issues	202 (20.7%)
Headache	603 (61.8%)
Loss of taste/smell	480 (49.2%)
Shortness of breath	451 (46.2%)
Trouble sleeping	276 (28.3%)
Most bothersome symptoms during course of COVID-19 (up to three selections)	
Body aches/joint pain	288 (29.5%)
Brain fog	106 (10.9%)
Cough	368 (37.7%)
Chest pain	162 (16.6%)
Fatigue	316 (32.4%)
Fever/chills	219 (22.4%)
GI issues	85 (8.7%)
Headache	232 (23.8%)
Loss of taste/smell	177 (18.1%)
Shortness of breath	212 (21.7%)
Trouble sleeping	52 (5.3%)
*Post-acute COVID-19 syndrome (“Long Covid”)	284 (29.1%)

*Participants with post-acute COVID-19 syndrome (PACS) self-identified as experiencing long COVID, they did not have to meet the current criteria for PACS definition.

**Fig 2:**
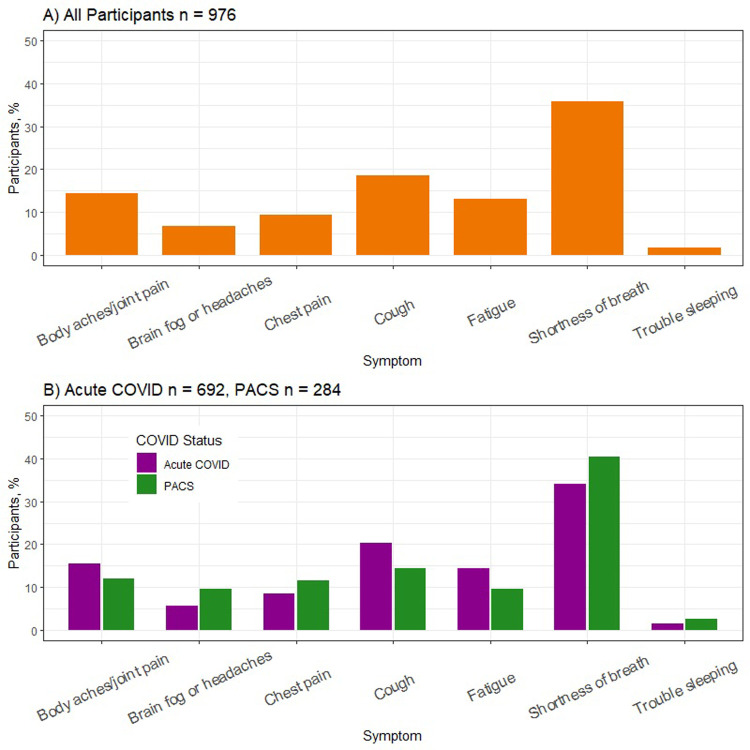
Symptoms that best described the COVID-19 experience for A) entire cohort B) by post-acute COVID-19 syndrome (PACS) status.

The ranking activity asked participants to rank which symptom best described their COVID-19 experience. The symptom most frequently reported in the position that best describes the COVID-19 experience was shortness of breath (36%) **(Fig 2A).**

### Participants with PACS

Of participants with self-reported PACS, 64.4% (183/284) had a time interval between their positive COVID-19 test and when they took the survey of 90 or more days which meets the current criteria for PACS [3]. Respondents with PACS were more likely to be, female, very religious, and to have required an emergency department visit or hospitalization during their COVID-19 infection **(Table 3).**

**Table 3:** Demographics by PACS status.

Demographic	Acute COVID-19 only, n = 692	PACS, n = 284	*p*-value
Age, years			0.331
18 to 34	229 (33.1%)	85 (29.9%)	
35 to 54	255 (36.8%)	119 (41.9%)	
55 plus	208 (30.1%)	80 (28.2%)	
**Gender: Female**	407 (58.8%)	190 (66.9%)	**0.021**
Race/Ethnicity			0.840
White	469 (67.8%)	190 (66.9%)	
Other	225 (32.5%)	95 (33.5%)	
Education			0.151
High school/GED	189 (27.3%)	95 (33.5%)	
Some College	227 (32.8%)	88 (31.0%)	
College Graduate	276 (39.9%)	101 (35.6%)	
Insurance: Insured	601 (86.8%)	249 (87.7%)	0.754
**Religiosity**			**0.007**
Not	109 (15.8%)	31 (10.9%)	
Somewhat	231 (33.4%)	78 (27.5%)	
Very	352 (50.9%)	175 (61.6%)	
**Care Level**			**≤0.001**
No Hospital	387 (55.9%)	102 (35.9%)	
Emergency Department	162 (23.4%)	97 (34.2%)	
Hospital Inpatient (includes ICU)	143 (20.7%)	85 (29.9%)	

The most common symptoms overall for the PACS group were similar to those reported by the entire cohort; however, symptoms of chest pain (49% compared to 32%; P<0.001), shortness of breath (57% compared to 42%; P<0.001), gastrointestinal issues (28% compared to 18%; P = 0.001), body aches/joint pains (73% compared to 63%; P = 0.006), trouble sleeping (33% compared to 26%; P = 0.04), and cognitive symptoms (46% compared to 25%; P<0.001), were reported disproportionately by respondents who reported PACS compared to those without PACS. Chest pain (24.6% compared to 13.3%; P < 0.001), GI symptoms (12.0% compared to 7.4% P = 0.03), and cognitive impairment (16.2% compared to 8.7%; P = 0.001), were reported most bothersome at higher rates by participants with PACS compared to those without PACS **(Table 4).**

**Table 4:** Symptoms by PACS status.

Demographic	Acute COVID-19 n = 692	PACS, n = 284	*p*-value
Symptoms over entire course of COVID-19 (multiple selections)			
**Body aches/joint pain**	436 (63.0%)	206 (72.5%)	**0.006**
**Brain fog (cognitive impairment)**	172 (24.9%)	131 (46.1%)	**<0.001**
**Chest Pain**	224 (32.4%)	138 (48.6%)	**<0.001**
Cough	486 (70.2%)	203 (71.5%)	0.756
Fatigue	506 (73.1%)	196 (69.0%)	0.223
Fever/chills	431 (62.3%)	177 (62.3%)	1.000
**GI issues**	123 (17.8%)	79 (27.8%)	**0.001**
Headache	419 (60.5%)	184 (64.8%)	0.244
Loss of taste/smell	327 (47.3%)	153 (53.9%)	0.071
**Shortness of breath**	290 (41.9%)	161 (56.7%)	**<0.001**
**Trouble sleeping**	182 (26.3%)	94 (33.1%)	**0.039**
Most bothersome symptoms during course of COVID-19 (up to three selections)			
Body aches/joint pain	202 (29.2%)	86 (30.3%)	0.793
**Brain fog (cognitive impairment)**	60 (8.7%)	46 (16.2%)	**0.001**
**Chest Pain**	92 (13.3%)	70 (24.6%)	**<0.001**
Cough	252 (36.4%)	116 (40.8%)	0.221
Fatigue	223 (32.2%)	93 (32.7%)	0.934
Fever/chills	156 (22.5%)	63 (22.2%)	0.970
**GI issues**	51 (7.4%)	34 (12.0%)	**0.028**
Headache	159 (23.0%)	73 (25.7%)	0.409
Loss of taste/smell	130 (18.8%)	47 (16.5%)	0.464
Shortness of breath	150 (21.7%)	62 (21.8%)	1.000
Trouble sleeping	43 (6.2%)	9 (3.2%)	0.077

Based on the ranking activity, the symptoms best describing the participants overall COVID-19 experience were different between participants with PACS and those without PACS (P<0.001). Participants with PACS reported shortness of breath at a higher rate and cough at a lower rate than non-PACS respondents **(Fig 2B)**.

### Coping profiles and patterns of coping

**Fig 3:**
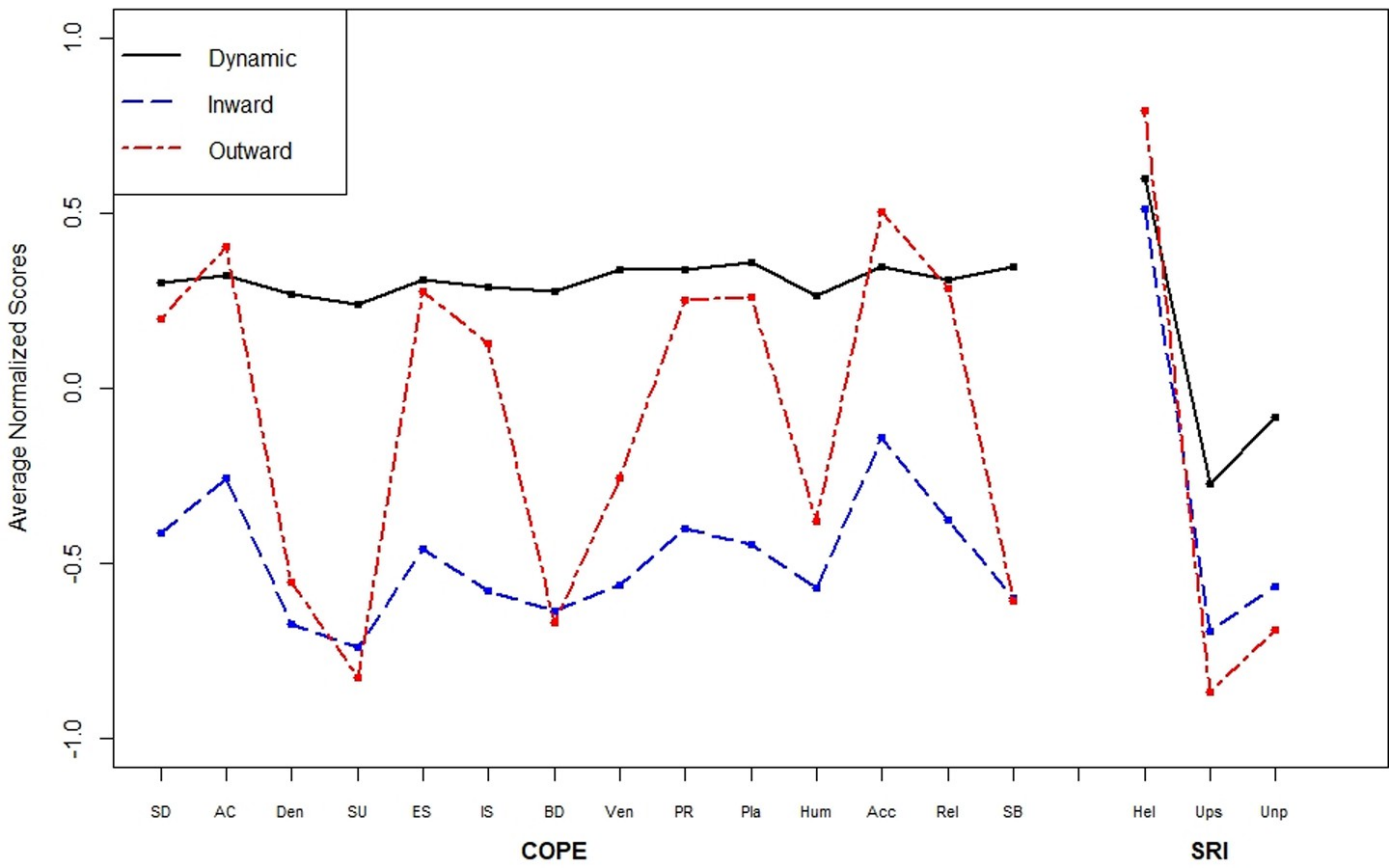
Normalized average scores for brief-COPE and SRI by cluster. Brief-COPE subscales include self-distracted (SD), active coping (AC), denial (Den), substance use (SU), emotional support (ES), instrumental support (IS), behavioral disengagement (BD), venting (Ven), positive reframing (PR), planning (Pla), humor (Hum), acceptance (Acc), religion (Rel), and self-blame (SB). Subscales for SRI include helpful (Hel), upsetting (Ups), and unpredictable (Unp).

Silhouette analysis based on misclassification rates from discriminant analysis identified three clusters as the optimal number of groups. **(ODS Fig 1 in S1 Data)** Principal component analysis indicated good separation between the three identified coping groups. **(ODS Fig 2 in S1 Data)** After discussion and consensus among the authors, and based on the cluster attributes, we named the three clustered groups (1) outward copers, (2) inward copers, and (3) dynamic copers further described below **(Fig 3).**

*Outward copers* (n = 284, 29%), when coping with COVID-19 illness stress these participants used more active coping strategies and reported receiving emotional and instrumental support from others. They reported higher levels of active coping (e.g., “I’ve been concentrating my efforts on doing something about the situation I’m in”) and drawing on their social networks for support. They also used relatively high levels of positive reframing, planning, humor, acceptance, and religion as coping strategies. This group reported relatively low levels of behavioral disengagement, substance use, or self-blame. In general, their coping could be described as very active, harnessing social support from others, and low in strategies associated with poorer mental or physical health (substance use, self-blame). They rated their close relationships as relatively high in helpfulness and low in upset or unpleasantness.

*Inward copers* (n = 358, 37%) when coping with COVID-19 illness stress, were less likely to receive emotional or instrumental support from others, and were also less likely to use planning, humor, or religion compared to other coping profiles.

The *dynamic copers* (n = 334, 34%) reported high levels of most coping strategies, both adaptive strategies such as acceptance, active coping, and less adaptive strategies such as substance use and denial. These patients reported more upset and unpleasantness in their relationships with close others compared to other profiles. These dynamic copers were using a broad variety of coping strategies.

### Coping profiles–Demographics and symptoms

Outward copers were characterized by female gender, white race, aged 55 plus, education beyond high school and very religious. Inward copers were nearly equally split between education levels. However, inward copers, also contained a higher proportion of patients reporting white race, those reporting no religiosity, and having received care outside of the hospital setting during their original illness. Dynamic copers when compared to inward and outward copers, were younger, male gender, belonged to a racial or ethnic minority, college graduates, and more likely to have visited the emergency department or be admitted to the hospital during their COVID-19 illness **(Table 5).**

**Table 5:** Demographics of coping profiles.

Demographic	Dynamic, n = 334	Inward, n = 358	Outward, n = 284	*p*-value
**Age, years**				**≤0.001**
18 to 34	156 (46.7%)	91 (25.4%)	67 (23.6%)	
35 to 54	149 (44.6%)	125 (34.9%)	100 (35.2%)	
55 plus	29 (8.7%)	142 (39.7%)	117 (41.2%)	
**Gender: Female**	173 (51.8%)	232 (64.8%)	192 (67.6%)	**≤0.001**
**Race/Ethnicity**				**≤0.001**
White	195 (58.4%)	269 (75.1%)	196 (69.0%)	
Other	141 (42.2%)	90 (25.1%)	90 (31.7%)	
**Education**				**≤0.001**
High school/GED	97 (29.0%)	115 (32.1%)	72 (25.4%)	
Some college	83 (24.9%)	130 (36.6%)	102 (35.9%)	
College Graduate	154 (46.1%)	113 (31.6%)	110 (38.7%)	
**Insurance: Insured**	278 (83.2%)	317 (88.5%)	255 (89.8%)	**0.034**
**Religiosity**				**≤0.001**
Not	34 (10.2%)	72 (20.1%)	34 (12.0%)	
Somewhat	117 (35.0%)	124 (34.6%)	68 (23.9%)	
Very	183 (54.8%)	162 (45.3%)	182 (64.1%)	
**Care Level**				**≤0.001**
No Hospital	125 (37.4%)	207 (57.8%)	157 (55.3%)	
Emergency Department	105 (31.4%)	97 (27.1%)	57 (20.1%)	
Hospital Inpatient	104 (31.1%)	54 (15.1%)	70 (24.6%)	

Symptoms also differed by coping profile. In selections of symptoms experienced (multiple selections permitted), the top five most common symptoms reported across all three coping profiles, although ranked differently by the profiles, were cough, body aches/joint pain, fatigue, fever/chills, and headache. When investigating symptom reports by profile, a higher proportion of outward copers reported symptoms in each of these 5 symptom groups (70%-88%) dynamic copers reported each of these 5 symptom groups in the lowest proportion (51%-60%), and inward copers reported each of these 5 symptoms groups at intermediate levels (61%-78%).

Dynamic copers reported that chest pain was a bothersome symptom at higher rates (25%) compared to outward or inward copers (P<0.001). A higher proportion of outward copers reported fatigue (39%) and shortness breath (27%) as bothersome compared to dynamic and inward copers. Dynamic copers reported PACS at a higher rate than either inward or outward copers (36%; p = 0.005) **(Table 6).**

**Table 6:** Symptoms by coping profiles.

Demographic	Dynamic, n = 334	Inward, n = 358	Outward, n = 284	*p*-value
Symptoms over entire course of COVID-19 (multiple selections)				
**Body aches/joint pain**	196 (58.7%)	237 (66.2%)	209 (73.6%)	**0.001**
**Brain fog**	80 (24.0%)	122 (34.1%)	101 (35.6%)	**0.002**
Chest Pain	133 (39.8%)	115 (32.1%)	114 (40.1%)	0.050
**Cough**	201 (60.2%)	258 (72.1%)	230 (81.0%)	**<0.001**
**Fatigue**	173 (51.8%)	279 (77.9%)	250 (88.0%)	**<0.001**
**Fever/chills**	174 (52.1%)	218 (60.9%)	216 (76.1%)	**<0.001**
**GI issues**	54 (16.2%)	77 (21.5%)	71 (25.0%)	**0.023**
**Headache**	172 (51.5%)	231 (64.5%)	200 (70.4%)	**<0.001**
**Loss of taste/smell**	135 (40.4%)	174 (48.6%)	171 (60.2%)	**<0.001**
**Shortness of breath**	133 (39.8%)	154 (43.0%)	164 (57.7%)	**<0.001**
**Trouble sleeping**	78 (23.4%)	104 (29.1%)	94 (33.1%)	**0.025**
Most bothersome symptoms during course of COVID-19 (up to three selections)				
Body aches/joint pain	98 (29.3%)	106 (29.6%)	84 (29.6%)	0.997
Brain fog	38 (11.4%)	44 (12.3%)	24 (8.5%)	0.279
**Chest Pain**	83 (24.9%)	38 (10.6%)	41 (14.4%)	**<0.001**
Cough	122 (36.5%)	137 (38.3%)	109 (38.4%)	0.860
**Fatigue**	88 (26.3%)	118 (33.0%)	110 (38.7%)	**0.004**
Fever/chills	68 (20.4%)	76 (21.2%)	75 (26.4%)	0.157
GI issues	30 (9.0%)	30 (8.4%)	25 (8.8%)	0.959
Headache	80 (24.0%)	86 (24.0%)	66 (23.2%)	0.969
Loss of taste/smell	57 (17.1%)	67 (18.7%)	53 (18.7%)	0.822
**Shortness of breath**	68 (20.4%)	66 (18.4%)	78 (27.5%)	**0.017**
Trouble sleeping	20 (6.0%)	17 (4.7%)	15 (5.3%)	0.768
**Post-acute COVID-19 syndrome**	119 (35.6%)	92 (25.7%)	73 (25.7%)	**0.005**

**Fig 4:**
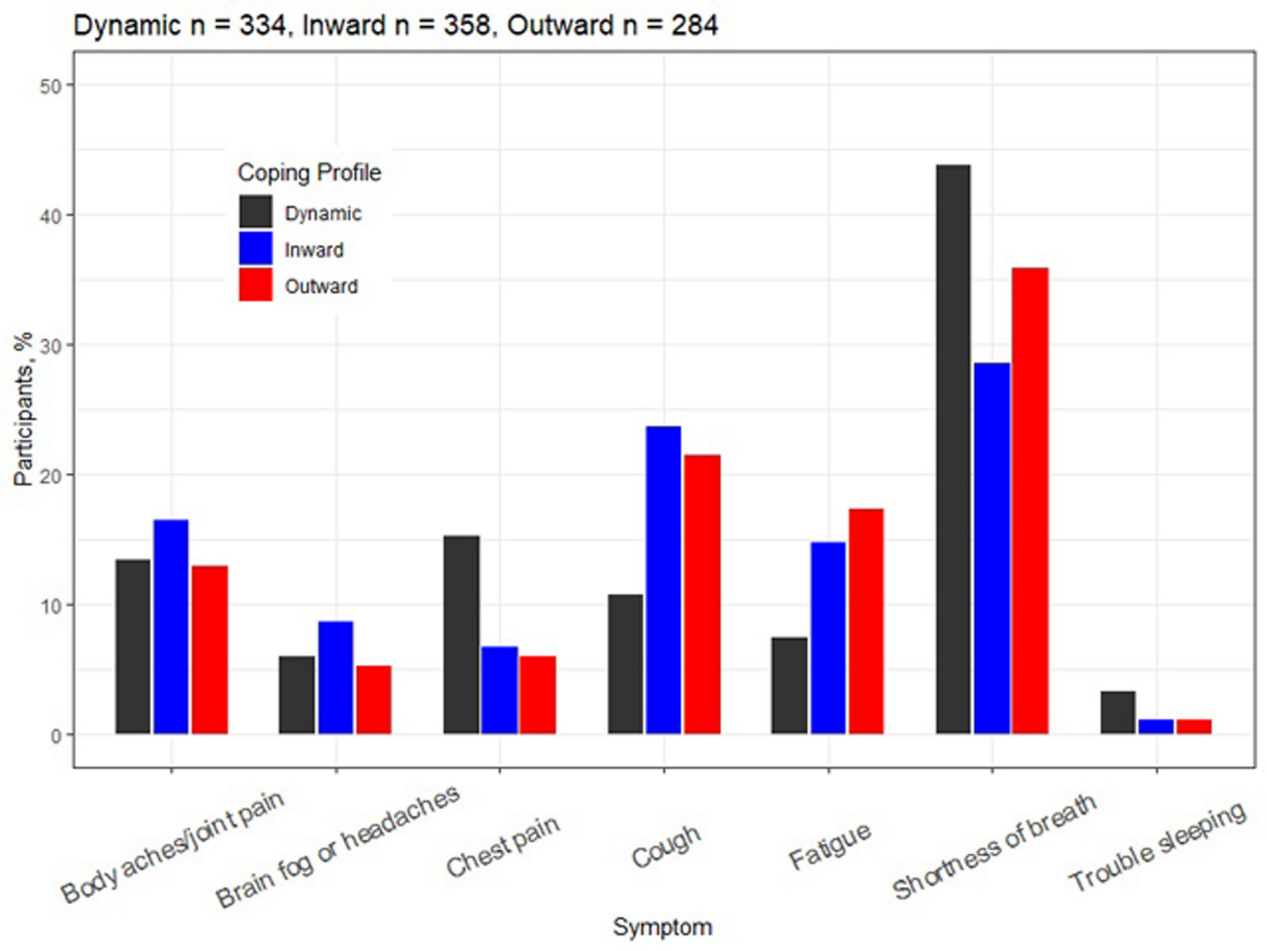
Symptoms that best described the COVID-19 experience by coping profiles.

All coping profiles ranked shortness of breath as the symptom that best defined their COVID-19 experience. Dynamic copers indicated that chest pain best characterized the COVID-19 experience at a higher rate than the other two profiles. However, inward and outward copers chose cough and fatigue at higher rates when compared to dynamic copers when asked about symptoms that described their COVID-19 experience **(Fig 4).**

## Discussion

This study contributes to the growing body of literature describing both acute and chronic symptoms of COVID-19 in an ethnically and racially diverse group of Americans. We uniquely centered the experience of COVID-19 around participant prioritized symptoms and explored how coping profiles related to the experience of COVID-19 disease and PACS. The most common symptoms reported by our diverse cohort were cough, fatigue, and body aches/joint pains. PACS was reported by 29% of our cohort and those who reported PACS were more likely to be bothered by chest pain and brain fog than participants without PACS. Importantly, participants with PACS were also more likely to report being hospitalized (including ICU) or visiting the emergency department than those who did not report PACS.

The most common symptoms overall for the PACS group were like those reported by the entire cohort; however, symptoms of gastrointestinal issues, trouble sleeping, and cognitive symptoms were all more likely to be reported by individuals with PACS than those without PACS. Interestingly, patients with PACS ranked fatigue as a bothersome symptom less frequently than those who reported acute COVID-19 recovery (i.e., those who did not report PACS). This is in contrast to other reports of non-hospitalized patients with PACS who reported anosmia, fatigue, and shortness of breath as persistent symptoms [32]. A recent Israeli study investigated outcomes of patients with mild COVID-19 and found anosmia, dysgeusia, memory impairment, dyspnea, and weakness were reported as long lasting symptoms [33]. In our study, patients with PACS were more likely to be admitted to the hospital. This suggests that severity of a person’s COVID-19 experience may shape the symptoms rated as most bothersome. Participants with PACS reported that shortness of breath was the most defining symptom of their COVID-19 experience at a higher rate when compared to the acute COVID-19 participants who rated several symptoms more equally as defining their overall COVID-19 experience. Women were disproportionately represented in the PACS group, as were respondents who reported being very religious. The relationship between the most bothersome COVID-19 symptoms and individual characteristics is unclear and remains an area for additional investigation.

We also found three distinct coping profiles (inward, outward, and dynamic) based on the Brief-COPE and the SRI and explored how coping profiles related to the COVID-19 experience. Symptom patterns were relatively similar across coping profiles with all profiles reporting the same top 5 symptom groups within the coping profile. Chest pain was disproportionately to be reported most bothersome by dynamic copers compared to the other two profiles. Overall, however, our findings appear to suggest that symptom patterns are more different by PACs vs. acute COVID-19 patients rather than by coping mechanism. It is important to note that in our cohort PACS is associated with more severe initial disease (based on hospitalization status). Alternatively, most recent reviews suggest that it is not clear whether acute disease severity is more or less likely to relate to PACS [34, 35].

Coping strategies include action-focused (taking action to change a situation, planning what to do next), emotion-focused (positively reframing the situation, using humor), and strategies sometimes described as harmful (blaming oneself, mental disengagement) [36, 37]. The outward copers had the arguably healthiest coping strategies. Hospitalization and fatigue are both associated with poor psychological outcomes, and poor psychological outcomes are often associated with more inward coping profiles [38]. Despite a disproportionate likelihood of fatigue compared to other profiles and of hospitalizations compared to inward copers, the outward copers marshaled healthy coping mechanisms indicative of positive action and planning.

The dynamic coping profile emerged as a new cluster of coping strategies not clearly described previously [27]. This group is interesting as they are more likely to be male, younger, and to be Black and Hispanic/Latino than the other two profiles. They activate many coping strategies that are both positive and negative. The dynamic coping group reported higher frequency of PACS than the patients in the other two coping profiles. Potentially, dynamic copers may be testing out different coping strategies to try and find one that works best to manage this new change to their lives. The COVID-19 illness may be one of the first, if not the only major health challenges they have faced and therefore this group may not have previously required or discovered clear coping mechanisms. In addition, cultural changes and the way general media, social media, and technology have evolved over the past few years may have changed the ways in which younger patients cope with illness [39]. Younger people coping with the pandemic may also exhibit more distress relating to the use of less adaptive coping strategies [40]. Furthermore, recent data about examining coping profiles longitudinally during the COVID-19 pandemic suggest coping strategy pattern changes may be relatively common across time [41].

In our prior work we report associations between coping profiles and decision making related to chronic illness [27]. In this study we extended this work to understand coping profiles in relation to COVID-19 experiences and reports of PACS. Coping mechanisms and individual characteristics can influence experiences with a disease and quality of life [42–44]. Dynamic copers, who were comparatively high on using maladaptive coping strategies (like substance use) were the coping group most likely to report having PACS. Outward copers had relatively high levels of hospitalization (compared to inward copers) but were using few maladaptive and more adaptive coping strategies. This is consistent with models of resilience and post-traumatic growth [45]. Additionally, outward copers were likely to be older and may have learned more adaptive strategies to marshal over time. Recent data examining coping specifically in patients who have COVID-19 are demonstrating that coping strategies for those with PACS may promote improved health agency [46]. In one study of patients hospitalized with COVID-19, indicating a relatively severe acute illness, participants stated that COVID-19 was often a health turning point, and that a sense of profound gratitude, a change in outlook, and engagement in healing behaviors (e.g., exercise) accompanied recovery from COVID-19 [47]. This is supported in our data by outward and dynamic copers use of adaptive coping strategies.

The association between coping profile and the self-reported experience of PACS is an area for additional investigation. The link between coping profiles and patient experience may help clinicians develop integrative treatments that are more patient rather than population centered. Much like genetic patterning and the body’s response to SARS-CoV-2, it is possible coping profiles contribute to a body’s physiologic response to disease, treatment, and experience.

Limitations to this study include those that are common to survey investigations. Our primary data collection and survey development occurred in the early stages of the COVID-19 pandemic and were focused on symptoms. Other important diseases, such as pulmonary embolism, were not addressed in this study. The results rely on the participant’s lived experience which may be affected by participation bias and social desirability; however, our large cohort is a representative US sample with a high response rate which minimizes bias. Second, the presence of PACS was not confirmed by qualified medical diagnostics and 36% of patients in our study did not meet the current diagnostic criteria for PACS. However, given the recent emergence of a formal definition of PACS and the imminent focus on patient-centered outcomes, the fact that some patients self-identified as having the syndrome is telling. This work is largely exploratory and may suffer from statistical limitations regarding multiple comparisons. Comparisons in the exploratory analysis are not definitive but do provide fertile ground for further investigations into the experience of PACS and individual traits like coping profiles.

## Conclusion

Cough, fatigue, and body aches/joint pain are consistently among the most important symptoms reported by patients with acute COVID-19 or PACS. Participants with PACS compared to those with acute COVID-19 reported GI symptoms and cognitive symptoms at disproportionately higher rates. Participants with PACS also ranked fatigue as the symptom that defined their disease experience less frequently and they reported shortness of breath as the most defining symptom of their COVID-19 experience. Three distinct coping profiles were used by participants in our cohort, outward coping, inward coping, and dynamic coping. Outward copers reported fatigue and cough at the highest rates yet were marshaling generally healthy coping strategies. Dynamic copers were unique as they reported many positive and negative coping mechanisms, were more likely to be young, male, Black, Hispanic/Latino, and more likely to self-report PACS. Further investigations into coping profiles in general, especially the dynamic coping profile, and the experience of COVID-19 related long term illness is warranted.

## Supporting information

S1 Checklist STROBE statement—checklist of items that should be included in reports of observational studies. (DOCX)

S1 Data (DOCX)

S2 Data (PDF)
