# Supplementary material for: Patient experiences with SARS-CoV-2: Associations between patient experience of disease and coping profiles
Source: PLoS One. 2023 Nov 20;18(11):e0294201. doi: 10.1371/journal.pone.0294201 (PMC10659202; doi:10.1371/journal.pone.0294201)
Supplement: S1 Data — (DOCX) [file pone.0294201.s002.docx]

**Online Data Supplement**

**Patient experiences with SARS-CoV-2: Associations between patient experience of disease and coping profiles**

**Online Survey Questions**

**See PDF**


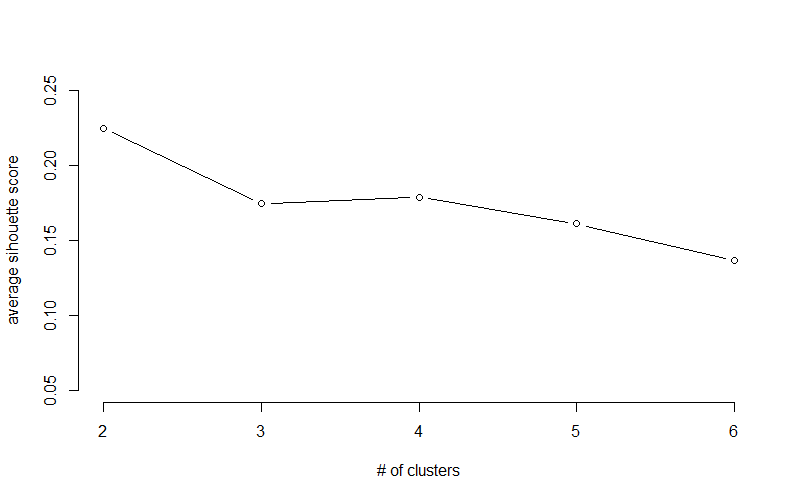


**ODS Figure 1:** Silhouette analysis of normalized Brief-COPE and SRI instruments while maintaining adequate group size indicates the optimal number of clusters is 3.


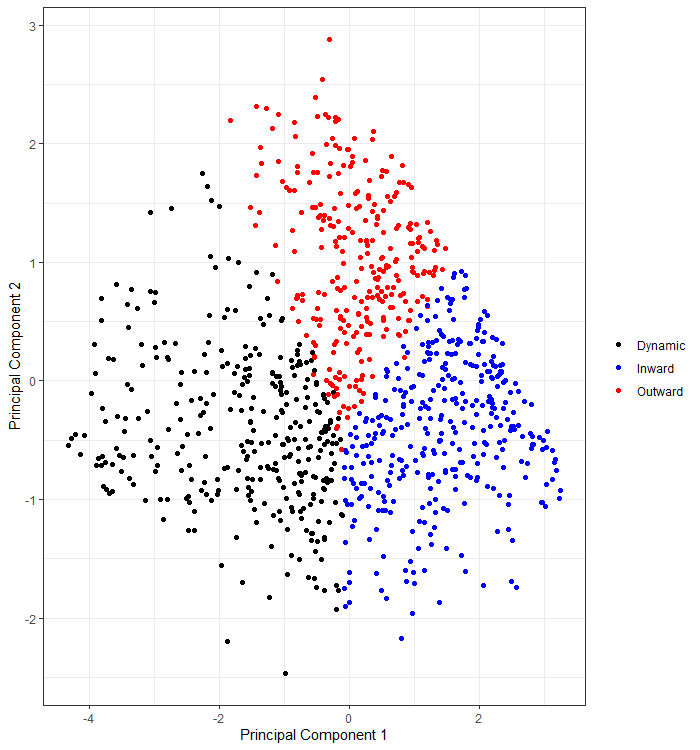


**ODS Figure 2:** Visualization of the first two components of the principal component analysis with respect to the three identified clusters indicates good separation.
