# Supplementary material for: Patient experiences with SARS-CoV-2: Associations between patient experience of disease and coping profiles
Source: PLoS One. 2023 Nov 20;18(11):e0294201. doi: 10.1371/journal.pone.0294201 (PMC10659202; doi:10.1371/journal.pone.0294201)
Supplement: S2 Data — (PDF) [file pone.0294201.s003.pdf]

Patient experiences with SARS-CoV-2: Associations between coping profiles and patient experience of disease.

Online Data Supplement
Questions from Online Survey

Q1. You are invited to participate in a survey about how to improve treatment methods for people who were infected with COVID-19. This survey will ask about your immediate and long-term symptoms and overall experience with COVID-19.

Your responses are anonymous. The survey will take about 15 minutes to complete.

By clicking on the arrow below to begin the survey, you are consenting to participate in this study. This study is voluntary, and you may stop at any time.

The COVID-19 pandemic has impacted all of us. As a country we now face new challenges, and we still do not know enough about the experience and recovery of people infected with COVID-19. By participating in this study, you are helping clinicians learn more about how we can better care for and treat people infected with this disease. We need your input so that we can create treatments that alleviate symptoms that matter most to YOU.

Together we can beat COVID-19.

Q2. Has a doctor, nurse, or other medical provider told you that you had COVID-19 since January 2021?

○ Yes

○ No

Q3. Did you complete a PCR test (which includes either a nasal swab or spitting into a container and waiting more than 2 hours) or have a positive result from a hospital or medical provider to confirm that you had COVID-19?

○ Yes

○ No

Q4. What was the date of the PCR test that confirmed you had COVID-19? Month (MM), Year (YYYY)

○ Month ____

○ Year ____

Q5. Did you ever seek medical care (this includes primary care visit, urgent care, emergency department, and/or telehealth) concerning COVID-19 (illness or recovery)?

- ○ Yes
- ○ No

Q6. Where did you seek medical care for your COVID-19 illness?

- ☐ Talked with a doctor through a virtual visit
- ☐ Clinic
- ☐ Urgent care
- ☐ Emergency Room/Hospital
- ☐ Pharmacy or drugstore ONLY
- ☐ Don’t know or don’t remember

Q7. **Thinking about your experiences early on with COVID-19…**

Q8. What was the **first** symptom you noticed? Select one or two

- ☐ Body aches or joint pain
- ☐ "Brain fog"
- ☐ Chest pain
- ☐ Cough
- ☐ Fatigue or feeling tired/weak
- ☐ Fever/chills/sweating
- ☐ GI issues such as vomiting, diarrhea
- ☐ Headache
- ☐ Loss of taste and/or smell
- ☐ Shortness of breath
- ☐ Trouble sleeping/insomnia
- ☐ Other: ____

Q9. **Over the entire course of your illness**, what symptoms did you have? *Check all that apply*

- ☐ Body aches or joint pain
- ☐ "Brain fog"
- ☐ Chest pain
- ☐ Cough
- ☐ Fatigue or feeling tired/weak
- ☐ Fever/chills/sweating
- ☐ GI issues such as vomiting, diarrhea
- ☐ Headache
- ☐ Loss of taste and/or smell
- ☐ Shortness of breath
- ☐ Trouble sleeping/insomnia
- ☐ Other: ____

Q10. What would you say were your **most bothersome or difficult symptoms** during the course of your COVID illness? *Select your top three*

- ☐ Body aches or joint pain
- ☐ "Brain fog"
- ☐ Chest pain
- ☐ Cough
- ☐ Fatigue or feeling tired/weak
- ☐ Fever/chills/sweating
- ☐ GI issues such as vomiting, diarrhea
- ☐ Headache
- ☐ Loss of taste and/or smell
- ☐ Shortness of breath
- ☐ Trouble sleeping/insomnia
- ☐ Other: ____

Q11. About how many days into your illness did you decide to seek medical care?

____

Q12. Briefly explain what symptoms or experiences made you (or a loved one) think you needed medical care:

This may be similar to the first symptoms you noticed, and that's okay.

____

Q13. Did you ever go to the emergency room or hospital when you had COVID-19?

- ◯ Yes
- ◯ No

Q14. **Thinking about your experiences in the hospital with COVID-19...**

Q15. What symptoms or outcomes were you most worried about during your hospital stay?

____

Q16. What level of care did you need in the hospital?

- ☐ Emergency room
- ☐ General hospital floor or unit
- ☐ ICU

Q17. What would you say were your most bothersome, difficult, or memorable symptoms during your hospital stay?

____
____
____
____
____

Q18. How many days were you in the hospital?

____

Q19. How, if at all, were you able to communicate with your loved ones while in the hospital? *Check all that apply*

- ☐ Did not communicate with family at all
- ☐ Telephone call
- ☐ Texting
- ☐ Video call
- ☐ My loved ones were with me in-person

Q20. Which communication method did you find most helpful to you? *Select your top choice*

- ○ Did not communicate with family at all
- ○ Telephone call
- ○ Texting
- ○ Video call
- ○ In-person

Q21. What specific medications or treatments, if any, do you remember having while in the hospital?

- [ ] Anticoagulation drugs ("blood thinners")
- [ ] Convalescent plasma
- [ ] Fluvoxamine
- [ ] Hydroxychloroquine
- [ ] Ivermectin
- [ ] Monoclonal antibody treatments (Tocilizumab)
- [ ] Remdesivir
- [ ] Steroids (Dexamethasone/ Methylprednisolone/ Prednisone)
- [ ] Don’t know or don’t remember
- [ ]

Q22. What type of oxygen, if any, did you need while in the hospital?

- [ ] No oxygen
- [ ] Regular nose oxygen
- [ ] Face-mask oxygen
- [ ] High flow nose oxygen
- [ ] CPAP or BIPAP
- [ ] Artificial breathing machine (intubation and a ventilator)
- [ ] Don't know or don't remember

Q23. Were you discharged from the hospital with oxygen?

- ○ Yes
- ○ No
- ○ Don't know or don't remember

Q24. Were you discharged home or to another facility?

- ○ Home
- ○ Skilled nursing or rehab facility
- ○ Other: ____

Q25. **Thinking about your experiences in urgent care or a clinic for COVID-19...**

This could be thinking back to your first visit with a medical provider, or during any type of follow-up assessment after a **hospitalization**.

Q26. What symptoms or outcomes were you most worried about when you went to urgent care or to see a medical provider in a clinic?

____

Q27. How many times have you gone back to visit urgent care or a clinic for your COVID-19 illness and recovery?

- ○ Once more
- ○ 2-3 times
- ○ 4-5 times
- ○ 6+ times
- ○ None, I did not go back for more care

Q28. Please briefly explain what symptoms or experiences made you (or a loved one) think you needed to go back for more medical care:

____

Q29. Below you will find a list of routine, daily activities. We would like you to rank them in order of 1 = describes my experience while I had COVID-19 extremely well to 7 = does not describe my experience at all.

To rank the items, simply click to drag and drop in the order that describes your experience. Remember there are no right or wrong answers.

During your illness and recovery, were you able to...
______ Think and speak clearly (brain fog)
______ Care for a child, spouse, or loved one
______ Get around your home and caring for oneself (shower, climbing stairs, etc.)
______ Cook and clean for oneself
______ Exercise
______ Return to work
______ Function without medical equipment such as oxygen, etc.

Q30. Now, rank by **how important** this daily activity is or was for you to achieve during recovery from COVID-19, from 1 = extremely important to 7 = not at all important.

During your recovery from COVID-19, how important was it for you to be able to...
______ Think and speak clearly (brain fog)
______ Care for a child, spouse, or loved one
______ Get around your home and caring for oneself (shower, climbing stairs, etc.)
______ Cook and clean for oneself
______ Exercise
______ Return to work
______ Function without medical equipment such as oxygen, etc.

Q31. Rank the following **symptoms** in relation to your experience with COVID-19 in order from 1 = describes my experience extremely well to 7 = does not describe my experience at all.

Did you have...
______ Shortness of breath
______ Chest pain
______ Body aches or joint pain
______ Cough
______ Fatigue or feeling tired/weak
______ Cognitive issues such as "brain fog" or headaches
______ Trouble sleeping or insomnia

Q32. Rank by **how important** it was for you to be free from the following symptoms during recovery from COVID-19, from 1 = extremely important to 7 = not at all important.

How important was it for you to be free from...
______ Shortness of breath
______ Chest pain
______ Body aches or joint pain
______ Cough
______ Fatigue or feeling tired/weak
______ Cognitive issues such as "brain fog" or headaches
______ Trouble sleeping or insomnia

Q33. Rank the following **feelings** in relation to your experience with COVID-19 in order from 1 = describes my experience extremely well to 7 = does not describe my experience at all.

Did you feel...
______ Anxious
______ Alone or isolated
______ Frustrated with the time it took to recover
______ Depressed
______ Lack of motivation
______ Fearful to do the things I was doing before
______ Unsupported or not believed in your illness journey

Q34. Rank by **how important** it was for you to achieve the following feelings during recovery from COVID-19, from 1 = extremely important to 7 = not at all important.

How important was it for you to be free from feeling...
______ Anxious
______ Alone or isolated
______ Frustrated with the time it took to recover
______ Depressed
______ Lack of motivation
______ Fearful to do the things I was doing before
______ Unsupported or not believed in your illness journey

Q35. Overall, how would you describe your physical health prior to your COVID-19 illness?

- ○ Poor
- ○ Fair
- ○ Good
- ○ Very good
- ○ Excellent

Q36. Prior to your COVID-19 illness, did you have any of the following respiratory ailments?

- ☐ Asthma
- ☐ Chronic hypoxemia
- ☐ COPD
- ☐ None

Q37. Prior to your COVID-19 illness, were you being treated for any of the following chronic medical conditions?

- ☐ Cancer
- ☐ Diabetes
- ☐ Heart disease
- ☐ High blood pressure
- ☐ Immunosuppressed
- ☐ Lung disease (excluding those mentioned in previous question)
- ☐ Stroke
- ☐ Other: ____
- ☐ None

Q38. Do you consider yourself as someone with “long COVID” or “long haulers syndrome”?

- ◯ Yes
- ◯ No

Q39. Can you briefly describe why you consider yourself as someone with “long COVID” or “long haulers syndrome”?

____

Q40. Are you able to be as active now as you were prior to your COVID-19 illness?

- ◯ Yes, definitely
- ◯ Yes, but with some limitations
- ◯ No

Q41. Are you experiencing any new anxiety, PTSD, depression, or other mental health concerns since your COVID-19 illness?

- ◯ Yes, definitely
- ◯ Yes, somewhat
- ◯ No

Q42. Are you experiencing any consistent changes to your sleep patterns since your COVID-19 illness?

- ☐ Yes, I sleep more
- ☐ Yes, I sleep less
- ☐ Yes, my sleep is poorer quality
- ☐ Yes, I now need medications to fall asleep
- ☐ No changes

Q43. In one word, how would you describe your experience with the COVID-19 illness?

____

Q44. Prior to your COVID-19 illness, were you vaccinated?

- ◯ Yes, received one dose of J&J or two doses of Moderna or Pfizer prior to illness
- ◯ Yes, received one dose of Moderna or Pfizer prior to illness
- ◯ No

Q45. To what extent did you engage in the following behaviors prior to your COVID-19 illness?

	Never	Sometimes	About half the time	Most of the time	Always
Smoking	◯	◯	◯	◯	◯
Socially distancing	◯	◯	◯	◯	◯
Visiting my doctor for an annual check-up	◯	◯	◯	◯	◯
Wearing a mask in public	◯	◯	◯	◯	◯
Wearing a mask in settings outside my personal home	◯	◯	◯	◯	◯
Wearing a seat belt	◯	◯	◯	◯	◯
Washing hands for 20 seconds frequently	◯	◯	◯	◯	◯

Q46. Which answer best describes your own state of health **TODAY.** First, think about your mobility. Would you say you...

- ◯ Have no problems in walking about
- ◯ Have some problems in walking about
- ◯ Are confined to your bed

Q47. Think about your self-care. Would you say you...

- ◯ Have no problems with self-care
- ◯ Have some problems washing or dressing yourself
- ◯ Are unable to wash or dress yourself

Q48. Next, think about your usual activities, for example work, study, housework, family or leisure activities. Would you say you...

- ◯ Have no problems with performing your usual activities
- ◯ Have some problems with performing your usual activities
- ◯ Are unable to perform your usual activities

Q49. Think about any pain or discomfort you experience. Would you say you have. . .

- ◯ No pain or discomfort
- ◯ Moderate pain or discomfort
- ◯ Extreme pain or discomfort

Q50. Finally think about your about anxiety or depression TODAY. Would you say you are. . .

- ◯ Not anxious or depressed
- ◯ Moderately anxious or depressed
- ◯ Extremely anxious or depressed

Q51. Looking at the scale below, imagine it is a thermometer where you can visualize the state of your health. The best state you can imagine is marked 100 at the top of the scale and the worst state you can imagine is marked zero at the bottom.

Where on this scale would mark your own state of health TODAY?

	Extremely bad Somewhat good Extremely good<br>0 10 20 30 40 50 60 70 80 90 100
Your own state of health today	

Q52. Have you **ever** been treated for anxiety by a physician or mental health provider?

- ○ Yes
- ○ No
- ○ Unsure

Q53. Have you **ever** been treated for depression by a physician or mental health provider?

- ○ Yes
- ○ No
- ○ Unsure

Q54. Please respond to the following statements, indicating to what extent you've been doing them to help you deal with the COVID-19 pandemic.

Take your time reading these questions before responding.

	I haven't been doing this at all	A little bit	A medium amount	I've been doing this a lot	A great deal
I've been turning to work or other activities to take my mind off things.	○	○	○	○	○
I've been concentrating my efforts on doing something about the situation I'm in.	○	○	○	○	○
I've been saying to myself "this isn't real".	○	○	○	○	○
I've been using alcohol or other drugs to make myself feel better.	○	○	○	○	○
I've been getting emotional support from others.	○	○	○	○	○
I've been giving up trying to deal with it.	○	○	○	○	○

I've been taking action to try to make the situation better.	○	○	○	○	○
I've been refusing to believe that it has happened.	○	○	○	○	○
I've been saying things to let my unpleasant feelings escape	○	○	○	○	○
I've been getting help and advice from other people.	○	○	○	○	○
I've been using alcohol or other drugs to help me get through it.	○	○	○	○	○
I've been trying to see it in a different light, to make it seem more positive.	○	○	○	○	○
I've been criticizing myself.	○	○	○	○	○
I've been trying to come up with a strategy about what to do.	○	○	○	○	○
I've been getting comfort and understanding	○	○	○	○	○

from someone.					
I've been giving up the attempt to cope.	○	○	○	○	○
I've been looking for something good in what is happening.	○	○	○	○	○
I've been making jokes about it.	○	○	○	○	○
I've been doing something to think about it less, such as going to movies, watching TV, reading, daydreaming, sleeping, or shopping.	○	○	○	○	○
I've been accepting the reality of the fact that it has happened.	○	○	○	○	○
I've been expressing my negative feelings.	○	○	○	○	○
I've been trying to find comfort in my religion or spiritual beliefs.	○	○	○	○	○
I've been trying to get	○	○	○	○	○

advice or help from other people about what to do.					
I've been learning to live with it.	○	○	○	○	○
I've been thinking hard about what steps to take.	○	○	○	○	○
I've been blaming myself for things that happened.	○	○	○	○	○
I've been praying or meditating.	○	○	○	○	○
I've been making fun of the situation.	○	○	○	○	○

Q55. When we need support such as advice, understanding or a favor, our **relationship with other people** may or may not have both helpful and upsetting aspects.

Think about the person **most important** to you (for example, parents, spouses, friends). Now, as you think about this person, please complete the following questions:

Q56. Thinking of the person most important to you, what is their relationship to you?

- ○ Grandparent
- ○ Parent
- ○ Spouse or life partner
- ○ Romantic partner
- ○ Child
- ○ Friend
- ○ Other ____

Q57. What is the gender of the person most important to you?

- ○ Female
- ○ Male
- ○ Non-binary / third gender

Q58. Approximate length of time you have known the person (in years):

____

Q59. Average number of times per week you have contact with the person:

____

Q60. In general, how helpful is the person to you?

- Not at all helpful
- A little helpful
- Somewhat helpful
- Moderately helpful
- Very helpful
- Extremely helpful

Q61. How upsetting is this person to you?

- Not at all upsetting
- A little upsetting
- Somewhat upsetting
- Moderately upsetting
- Very upsetting
- Extremely upsetting

Q62. How unpredictable is this person to you?

- Not at all unpredictable
- A little unpredictable
- Somewhat unpredictable
- Moderately unpredictable
- Very unpredictable
- Extremely unpredictable

Q63. How would you prefer to make decisions with your doctor?

- ◯ I prefer to make the final decision about which treatment I will receive
- ◯ I prefer to make the final decision about my treatment after seriously considering my doctor's opinion
- ◯ I prefer that my doctor and I share responsibility for deciding which treatment is best for me
- ◯ I prefer that my doctor makes the final decision about which treatment will be used, but seriously considers my opinion
- ◯ I prefer to leave all decisions regarding my treatment to my doctor

Q64. What is your age?

- ◯ 18-24
- ◯ 25-34
- ◯ 35-44
- ◯ 45-54
- ◯ 55-64
- ◯ 65+

Q65. What is your gender?

- ◯ Female
- ◯ Male
- ◯ Non-binary / third gender

Q66. Are you Hispanic, Latino/a, or Spanish origin?

- ◯ Yes
- ◯ No

Q67. What is your race? (One or more categories may be selected)

- ☐ American Indian or Alaska Native
- ☐ Asian
- ☐ Black or African American
- ☐ Native Hawaiian or Pacific Islander
- ☐ White
- ☐ Other (please specify) ____

Q68. In which state do you currently reside?

- ○ Alabama
- ○ Alaska
- ○ Arizona
- ○ Arkansas
- ○ California
- ○ Colorado
- ○ Connecticut
- ○ Delaware
- ○ District of Columbia
- ○ Florida
- ○ Georgia
- ○ Hawaii
- ○ Idaho
- ○ Illinois
- ○ Indiana
- ○ Iowa
- ○ Kansas

- Kentucky
- Louisiana
- Maine
- Maryland
- Massachusetts
- Michigan
- Minnesota
- Mississippi
- Missouri
- Montana
- Nebraska
- Nevada
- New Hampshire
- New Jersey
- New Mexico
- New York
- North Carolina
- North Dakota
- Ohio
- Oklahoma
- Oregon
- Pennsylvania
- Puerto Rico
- Rhode Island
- South Carolina
- South Dakota

- Tennessee
- Texas
- Utah
- Vermont
- Virginia
- Washington
- West Virginia
- Wisconsin
- Wyoming
- I do not reside in the United States

Q69. What is your ZIP code?

____

Q70. What is the highest level of school you have completed or the highest degree you have received?

- Less than high school degree
- High school graduate (high school diploma or equivalent including GED)
- Some college but no degree
- Associate degree in college (2-year)
- Bachelor's degree in college (4-year)
- Master's degree
- Doctoral degree
- Professional degree (JD, MD)

Q71. Are you a healthcare professional?

- ◯ Yes
- ◯ No

Q72. Did you have health insurance at the time of your COVID-19 illness?

- ◯ Yes
- ◯ No

Q73. How important is religion in your daily life?

- ◯ Not at all
- ◯ Slightly
- ◯ Somewhat
- ◯ Very
- ◯ Extremely important

Q74. How often do you attend religious services?

- ◯ Never
- ◯ Occasionally, for significant holidays or events
- ◯ Once a week
- ◯ 2-3 times per week
- ◯ 4-6 times per week
- ◯ Daily

Q75. What religious group do you belong to or identify yourself most close to?

- ◯ Buddhist
- ◯ Catholic
- ◯ Christian, other not listed
- ◯ Church of Jesus Christ of Latter-day Saints
- ◯ Hindu
- ◯ Jewish
- ◯ Muslim
- ◯ Protestant (such as Baptist, Methodist, Presbyterian)
- ◯ Spiritual but not religious
- ◯ Native American
- ◯ Not religious
- ◯ Other ____
